# PD142 Implantable Neurostimulation Devices For The Treatment Of Drug-Resistant Pediatric Epilepsy

**DOI:** 10.1017/S0266462324003787

**Published:** 2025-01-07

**Authors:** Vanesa Ramos García, Yolanda Álvarez-Pérez, Analía Abt Sacks, Amado Rivero-Santana, Andrea Duarte-Díaz, Anthea Santos-Álvarez, Yadira González-Hernández, Francisco Rivas-Ruiz, Lilisbeth Perestelo-Pérez

## Abstract

**Introduction:**

Epilepsy affects approximately 10.5 million individuals under the age of 15 years worldwide. In Spain, 3.7 per 1,000 inhabitants aged 6 to 14 years have epilepsy, making it the third most common neurological emergency. Drug resistance is observed in eight to 33 percent of cases. Responsive neurostimulation (RNS) systems could improve seizure control in pediatric patients who are not eligible for brain surgery.

**Methods:**

We systematically searched for articles published up to September 2022 in the following bibliographic databases: MEDLINE, Embase, Web of Science, and CINAHL. We included primary experimental and observational studies as well as case series studies addressing the safety, efficacy, and cost effectiveness of RNS in the treatment of drug-resistant pediatric epilepsy.

**Results:**

Two systematic reviews of prospective and retrospective case series studies and four primary experimental studies were identified. The case series studies found that a large proportion of pediatric patients responded to RNS, with a reduction of between 50 and 75 percent in the frequency of seizures. The intensity and duration of seizures also decreased after using RNS. Adverse effects of the RNS implantation process were related to infections, erythema, and hematomas. Only one study (n=17) reported moderate adverse effects related to stimulation (dysesthetic pain in the upper and lower right limb), but there were no serious reactions leading to RNS discontinuation.

**Conclusions:**

Randomized controlled trials in pediatric drug-resistant populations ineligible for brain surgery with adequate sample sizes are needed to determine the effectiveness of RNS in terms of seizure frequency, duration, and intensity. No cost-effectiveness studies have been conducted on RNS in this cohort.

